# Evaluating the lettuce metatranscriptome with MinION sequencing for future spaceflight food production applications

**DOI:** 10.1038/s41526-021-00151-x

**Published:** 2021-06-17

**Authors:** Natasha J. Haveman, Christina L. M. Khodadad, Anirudha R. Dixit, Artemis S. Louyakis, Gioia D. Massa, Kasthuri Venkateswaran, Jamie S. Foster

**Affiliations:** 1 Department of Microbiology and Cell Science, University of Florida, Space Life Science Lab, Merritt Island, FL USA; 2 Amentum Services, Inc., LASSO, Kennedy Space Center, Merritt Island, FL USA; 3 Department of Molecular and Cell Biology, University of Connecticut, Storrs, CT USA; 4 Space Crop Production Team, Kennedy Space Center, Merritt Island, FL USA; 5 Biotechnology and Planetary Protection Group, Jet Propulsion Laboratory, Pasadena, CA USA

**Keywords:** Microbiology, Plant sciences

## Abstract

Healthy plants are vital for successful, long-duration missions in space, as they provide the crew with life support, food production, and psychological benefits. The microorganisms that associate with plant tissues play a critical role in improving plant health and production. To that end, we developed a methodology to investigate the transcriptional activities of the microbiome of red romaine lettuce, a key salad crop that was grown under International Space Station (ISS)-like conditions. Microbial transcripts enriched from host–microbe total RNA were sequenced using the Oxford Nanopore MinION sequencing platform. Results show that this enrichment approach was highly reproducible and could be an effective approach for the on-site detection of microbial transcriptional activity. Our results demonstrate the feasibility of using metatranscriptomics of enriched microbial RNA as a potential method for on-site monitoring of the transcriptional activity of crop microbiomes, thereby helping to facilitate and maintain plant health for on-orbit space food production.

## Introduction

As humanity strives to prepare for the upcoming manned missions to the Moon and *cis*-lunar orbit, it has become evident that growing plants for food and other life support systems is essential to humankind’s space exploration agenda^1–4^. To ensure that these future missions are successful, it is necessary to investigate all aspects of the basic physiological and molecular responses of food crops and their microbiomes to the spaceflight environment, so that any issues faced on these missions can be efficiently managed and mitigated on-orbit.

Plants have complex and dynamic relationships with their microorganisms, which are influenced by various stresses and environmental factors, including spaceflight^5–9^. The novel environments of spaceflight and microgravity analogs have been shown to alter the plant–microbe interactions at both the genetic and physiological levels, oftentimes resulting in increased susceptibility to diseases^10–14^. For example, soybean seedlings grown in spaceflight are more susceptible to the colonization of fungal pathogen, *Phytophthora sojae*, when compared to ground controls^10^. In addition, simulated microgravity experiments have demonstrated that clinorotation reduces the growth and development of the legume *Medicargo truncatula* and significantly affects its symbiotic relationship with the rhizobium *Sinorhizobium meliloti* and arbuscular mycorrhizal fungi *Rhizophagus irregularis*^15^. Specifically, nodule size and number were reduced under clinorotation when compared to static controls^15^. Moreover, in another simulated microgravity experiment performed on random positioning machines, the endophytic bacterial diversity in wheat (*Triticum aestivum*) seedlings behaves differently under simulated microgravity when compared to normal gravity controls^14^. Specifically, some bacterial genera (e.g., *Pseudomonas*, *Paenibacillus*, and *Bacillus*) that can produce plant growth-promoting and antimicrobial substances, exhibit clear differences in relative abundance between microgravity and Earth gravity treatments^14^. However, research on how microgravity impacts plant–microbe interactions is still in its infancy. It is, therefore, critical to develop efficient methods for investigating plant–microbe interactions to expand our current knowledge base for future space exploration. The dynamic nature of plant–microbial interactions requires regular monitoring of a crop’s microbiome and its metabolic activities to assess plant health. These measures together with other surveillance techniques (e.g., hyperspectral imaging) will ensure that successful cultivation of crops on long-duration missions in space can be accomplished.

To date, plants have been successfully grown in space in multiple hardware systems, such as the Vegetable Production System (Veggie), Advanced Plant Habitat, and the LADA greenhouse^16^. For example, edible leafy crops have been safely produced in the Veggie hardware on the International Space Station (ISS) and consumed by crew members^17^. There have been, however, sporadic instances of contamination with opportunistic fungal pathogens. In a Veggie experiment (VEG-01C), *Zinnia hybrida* plants were infected by an opportunistic fungal pathogen, *Fusarium oxysporum*^18,19^. *F. oxysporum* is a ubiquitous group of soilborne fungi that are highly adaptable to different habitats and include non-pathogenic strains, plant pathogens, and even opportunistic human pathogens^20^. Pathogenic strains of *F. oxysporum* can cause Fusarium wilt in nearly every agriculturally important crop^21^. Some multi-host isolates in plants also have the potential to cause invasive fungal infections in immunocompromised mammals^22–25^. Even though the fungal infection on the *Zinnia* plants was likely due to a malfunction of a fan in the Veggie hardware^26^, it demonstrated the importance of characterizing the healthy basal microbiome of all plants cultivated in space.

To achieve this goal, methodologies are needed to regularly monitor changes in the microbial community during flight to potentially mitigate the rise of pathogenic taxa. Currently, the standard protocol for the investigation of plant–pathogen interactions aboard the ISS is to harvest the plant material, freeze it, and return it to Earth for analysis^27^. However, with future missions beyond Low Earth Orbit likely taking up to 6 months between resupply missions, it will be critical to develop faster, on-side methodologies to regularly monitor and assess plant–microbe health.

Here we present a method for using metatranscriptomics coupled with nanopore sequencing to characterize the plant microbiome and their functional activity from plant–microbe total RNA samples that could be feasibly completed on-orbit. Metatranscriptomics has emerged as an effective methodology to characterize the plant microbiome and evaluate its transcriptional activity^28^. Transcriptional surveys, among many others, enable the identification and assessment of the main drivers of changes in plant health^29–32^. However, to date, metatranscriptomic studies of plant microbiomes have all utilized Illumina sequencing platforms, which are not feasible for spaceflight applications due to their large up-mass, weight, and footprint. The pocket-sized, USB-powered nanopore MinION, sequencing platform, which weighs <100 g and is capable of single-molecule sequencing of native full-length DNA and RNA molecules^33^, has emerged as a viable sequence platform for spaceflight. In 2016, the nanopore-sequencing system was successfully used aboard the ISS for DNA analysis and compared to next-generation sequencing platforms, such as Illumina MiSeq and PacBio RSII^34^. Although the nanopore system has been successfully used for DNA applications, its use for metatranscriptomic analyses is only now emerging^35–38^ and has not yet been used to analyze the metatranscriptome of microbial transcripts from plant–microbe total RNA samples.

As a proof-of-concept, we have characterized the microbiome of red romaine lettuce grown under a baseline condition (i.e., ISS-like ground controls) using MinION-based metatranscriptomics to demonstrate that plants can be rapidly and effectively screened using this molecular approach. By developing reliable methodologies to regularly monitor and compare changes in the functional activity of the plant microorganisms over time, early detection of opportunistic pathogens can help inform mitigation strategies and provide important insight into agricultural practices aboard spacecraft to maximize crop production and ensure crew safety on long-duration missions.

## Results

### Successful enrichment of the microbial RNA fraction from total RNA

**Fig. 1:**
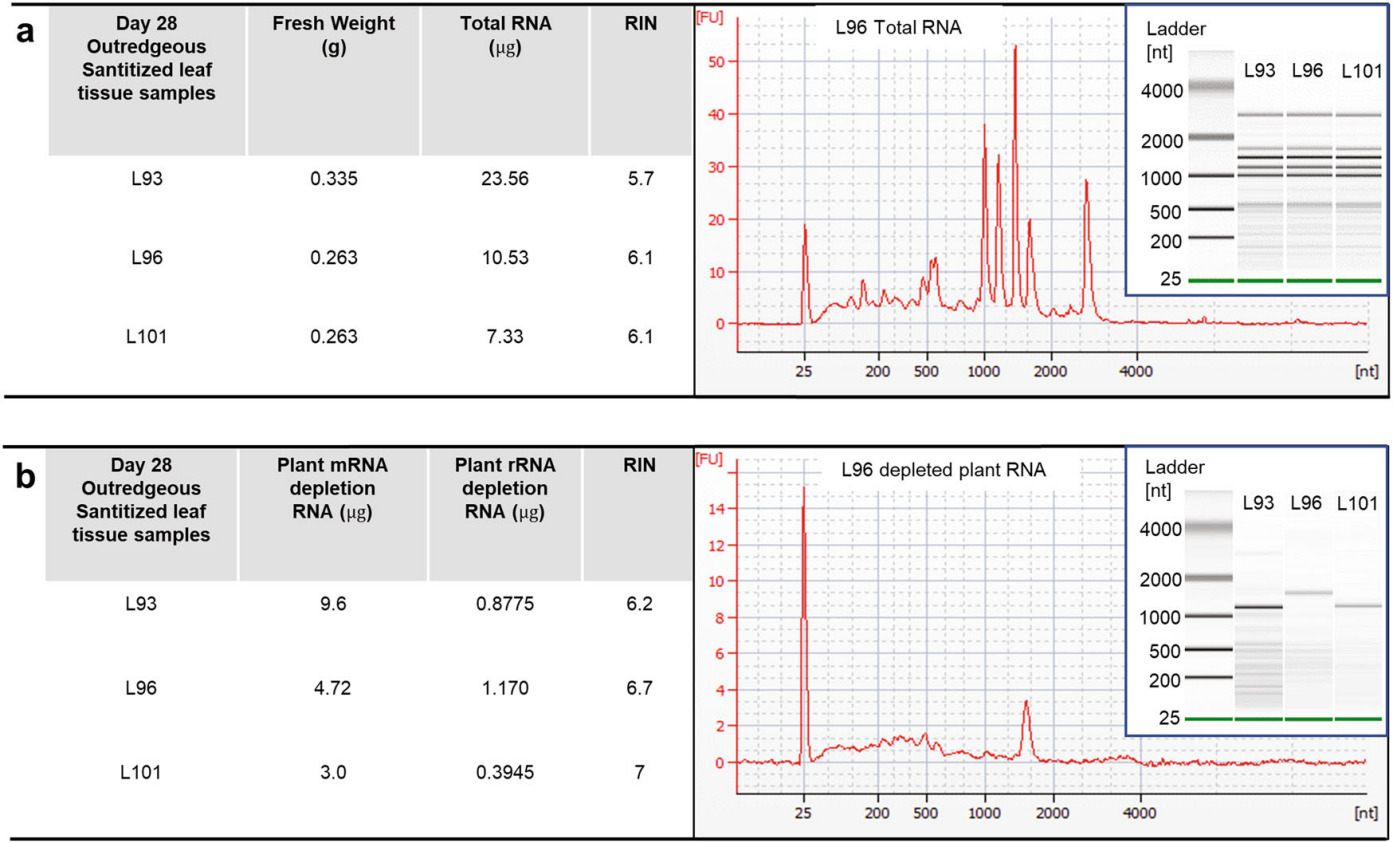
Qubit and Bioanalyzer analysis for enriched microbial RNA from host–microbial total RNA. **a** Quality and quantity of total RNA extracted from leaves of three individual 28-day-old Outredgeous lettuce plants (L93, L96, and L101). The table shows the amount of starting material used in the RNA extraction and total host–microbial RNA extracted for each sample. Bioanalyzer results of all three samples are shown in the digital gel image and a representative electropherogram from sample L96 shows the plant rRNA peaks. **b** The quality and quantity of the plant-depleted RNA were assessed. Bioanalyzer results of microbial enriched RNA are shown in the digital gel image and a representative electropherogram from sample L96 shows the microbial rRNA peak.

To ensure a comprehensive assessment of the microbes on the edible portion of the lettuce crop, both endophytic and epiphytic microbes of lettuce leaves were collected from plants grown in environmental chambers at the Kennedy Space Center (KSC) that mimicked conditions used aboard the ISS. Salad crops, such as lettuce, are important target crops for establishing a sustainable source of fresh food onboard a spacecraft, as they require minimal processing and can be consumed raw. The lettuce leaves were collected from three individual plants grown for 28 days in separate pots (denoted as L93, L96, and L101) each derived from a single surface-sanitized seed (see “Methods”)^39^. From each leaf, total RNA was extracted and then the microbial RNA fraction was enriched (Fig. 1a).

The high quality of the RNA derived from both the host and associated microbes is depicted in Fig.1a. Effective depletion of the host RNA was observed with the mean loss of 93% of the total RNA (L93, 96.28%; L96, 88.89%; L101, 94.62%), leaving only a residual ribosomal RNA (rRNA) peak for all replicates (Fig.1b). This percentage removal of host RNA was consistent with reports from similar studies that enriched for microbial RNA^40,41^, indicating that the newly developed enrichment protocol for bacterial messenger RNA (mRNA) was effective at removing a large quantity of plant transcripts.

### Overview of MinION-sequencing effectiveness of microbe-enriched metatranscriptome

The extracted RNA was converted to complementary DNA (cDNA), as currently the Oxford Nanopore direct RNA-sequencing kit does not yet enable barcoding and lower levels of starting template can be used. The replicate cDNA libraries were successfully run on individual MinION flowcells and generated a mean of 10,012,033 base-called reads per replicate pot (Table 1). A detailed figure of the bioinformatic pipeline used in this study is illustrated in Supplementary Fig. 1. After read processing and filtering, the L93, L96, and L101 samples consisted of 4,061,546 (58.34%), 10,730,313 (84.95%), and 7,361,010 (70.49%) high-quality reads, respectively (Table 1). The filtered reads were then mapped to the lettuce (*Lactuca sativa*) and closely related sunflower (*Helianthus annuus*) genomes, which are both well-characterized members of the *Asteraceae* family. All reads that mapped to either lettuce (L93, 19.17%; L96, 22.72%; L101, 7.23%) or sunflower (L93, 3.53%; L96, 5.52%; L101, 1.71%) were removed. Of the remaining reads, most encoded rRNAs (L93, 63.10%; L96 93.75%; L101, 69.34%) were removed for a separate taxonomic analysis. Of the rRNA transcripts, most were associated with the plant host and only the microbial 16S and non-host 18S rRNA reads were retained for taxonomic analysis with 521,345 (26.32%), 2,453,876 (33.99%), and 1,315,470 (28.31%) reads in L93, L96, and L101, respectively (Table 1).

**Table 1:** Summary statistics for metatranscriptome nanopore sequencing, mapping, and annotation for each sample.

	L93	L96	L101
Statistics for metatranscriptome sequences			
Mean read length (bp)	378.9	526.4	423.7
Read length N50 (bp)	417	616	512
Mean read quality (*Q*-score)	8	8.5	7
Percentage of reads > *Q*-score 5	86.20%	93.30%	81.80%
Total bases (bp)	2,637,799,078	6,649,786,942	4,424,479,269
Total number of reads	6,961,982	12,631,723	10,442,395
Total reads after trimming and QC^a^ (min length > 100 bp)	4,061,653	10,661,802	7,361,100
Filtered out host reads^b^	22.70%	28.24%	8.94%
Removed rRNA reads	63.10%	93.75%	69.34%
Metatranscriptome mapping and annotation results			
Reads used in taxonomy (16S and 18S)	521,345	2,453,876	1,315,470
Reads in non-rRNA analysis	1,158,293	481,186	2,055,342
Total Uniprot-annotated reads^c^ (*e*-value cutoff 0.05)	57,315	104,111	88,850
Uniprot other eukaryotic	9510	10,032	11,525
Uniprot bacteria and Archaea	2543	1238	4936
Uniprot fungi	780	346	1343
Uniprot viruses	253	108	538
Bacteria with KO annotation	2247	1075	4276
Fungi KO annotation	442	208	711

^a^Quality control (QC) threshold of *Q*-score < 5 and read length of <100 bp and >30,000 bp were excluded in the downstream analysis.

^b^Filtered out host reads—genomes used: GCF_002870075.1_Lsat_Salinas_v7_genomic.fna.gz (*L. sativa* (Lettuce)) and GCA_002127325.1_HanXRQr1.0_genomic.fna.gz (*H. annuus* (common sunflower)).

^c^Only Uniprot reads with *e-*value ≤ 0.05 were used for the metatranscriptomic analyses.

Of the remaining non-rRNA reads, only 57,315 (4.95%), 104,111 (21.64%), and 88,850 (4.32%) reads from the samples L93, L96, and L101, respectively, had an *e*-value cutoff of 0.05 similar to known proteins in the UniProt Swiss-Prot database (Table 1). Those microbial transcripts that could be annotated were further mapped against the Kyoto Encyclopedia of Genes and Genomes (KEGG) database to identify those functional genes with a KEGG Orthology (KO) identifier, resulting in the annotation of 2689 transcripts in L93, 1283 transcripts in L96, and 4987 transcripts in L101 (Table 1).

### Taxonomic composition of 28-day-old lettuce leaf microbial community

**Fig. 2:**
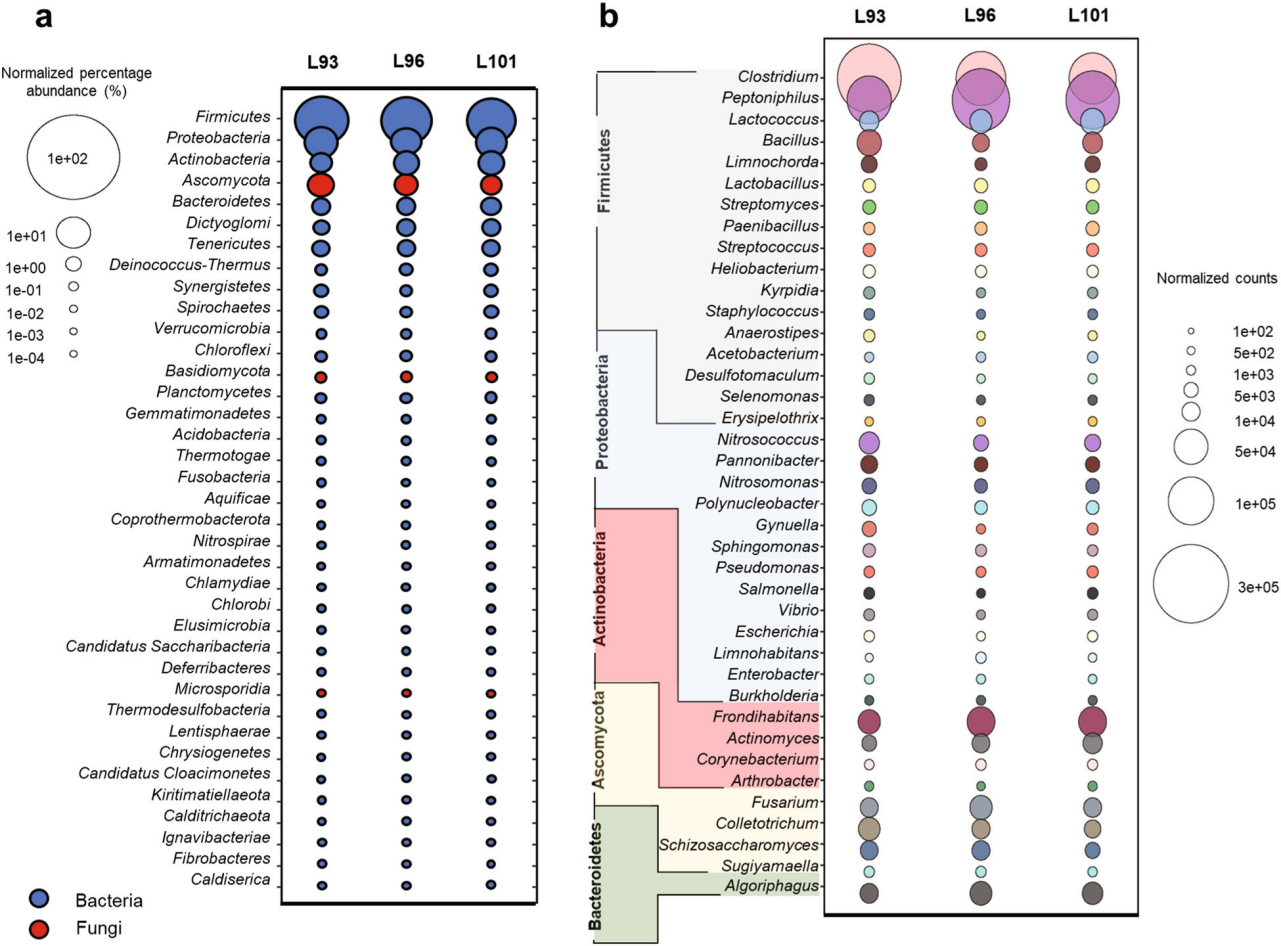
Taxonomy diversity of 16S and 18S rRNA reads in the leaf metatranscriptome samples analyzed by Kraken2. **a** Normalized percentage abundance of all bacterial and fungal phyla within each sample. Unclassified and Cyanobacteria were not included in the bubble plot. Bubble colors for each phylum that belong to bacteria and fungi are indicated by blue and red bubbles, respectively. The size of the bubble indicates the normalized percentage abundance of each phylum. **b** Bubble plots illustrating the normalized counts of genera from the top 5 phyla with ≥500 normalized read counts across all three samples. The size of the bubble indicates the normalized counts for each genus.

The transcriptionally active composition of the microbial communities and their relative abundance were characterized using the recovered 16S and non-host 18S rRNA transcripts to identify those taxa that associate with the lettuce leaf tissue. A total of 43 microbial phyla from all 3 microbial domains—Archaea, Bacteria, and Eukarya—were identified in these samples and are listed in Supplementary Dataset 1. Focus was placed on the recovered reads associated with bacteria and fungi, and the normalized percent abundances are illustrated in Fig. 2a. Due to the potential for misaligned chloroplast transcripts, all reads that were unclassified or assigned to the Cyanobacteria phylum were excluded from the taxonomic analyses. Of the five most abundant microbial phyla in the lettuce leaf microbiomes, four were bacterial (Firmicutes, Proteobacteria, Actinobacteria, and Bacteroidetes) and one was fungal (Ascomycota), which together comprised 15.48% of the community in sample L93, 13.13% in sample L96, and 15.04% in sample L101 (Supplementary Dataset 1).

Within these five most abundant phyla, a total of 1240 genera were identified (Supplementary Dataset 1); however, only those genera that had at least 500 normalized reads in all three samples were depicted in Fig. 2b. For each genus, the percentage listed was calculated from the total number of reads for that genus across all three samples relative to the total number of reads across all samples. The top ten most abundant genera within the top five phyla included *Clostridium* (31.89%), *Peptoniphilus* (29.28%), *Frondihabitans* (5.74%), *Lactococcus* (3.64%), *Bacillus* (3.08%), *Algoriphagus* (2.96%), *Fusarium* (2.68%), *Colletotrichum* (2.43%), *Actinomyces* (1.91%), and *Nitrosococcus* (1.88%) (Fig. 2b). Together, the top eight transcriptionally active bacterial genera represented between 78.34% and 81.67% of the recovered sequences in all three replicates, whereas the two fungal genera *Fusarium* and *Colletotrichum* comprised between 4.04% and 5.96% (Fig. 2b).

### Functional activity of the lettuce leaf microbial community

**Fig. 3:**
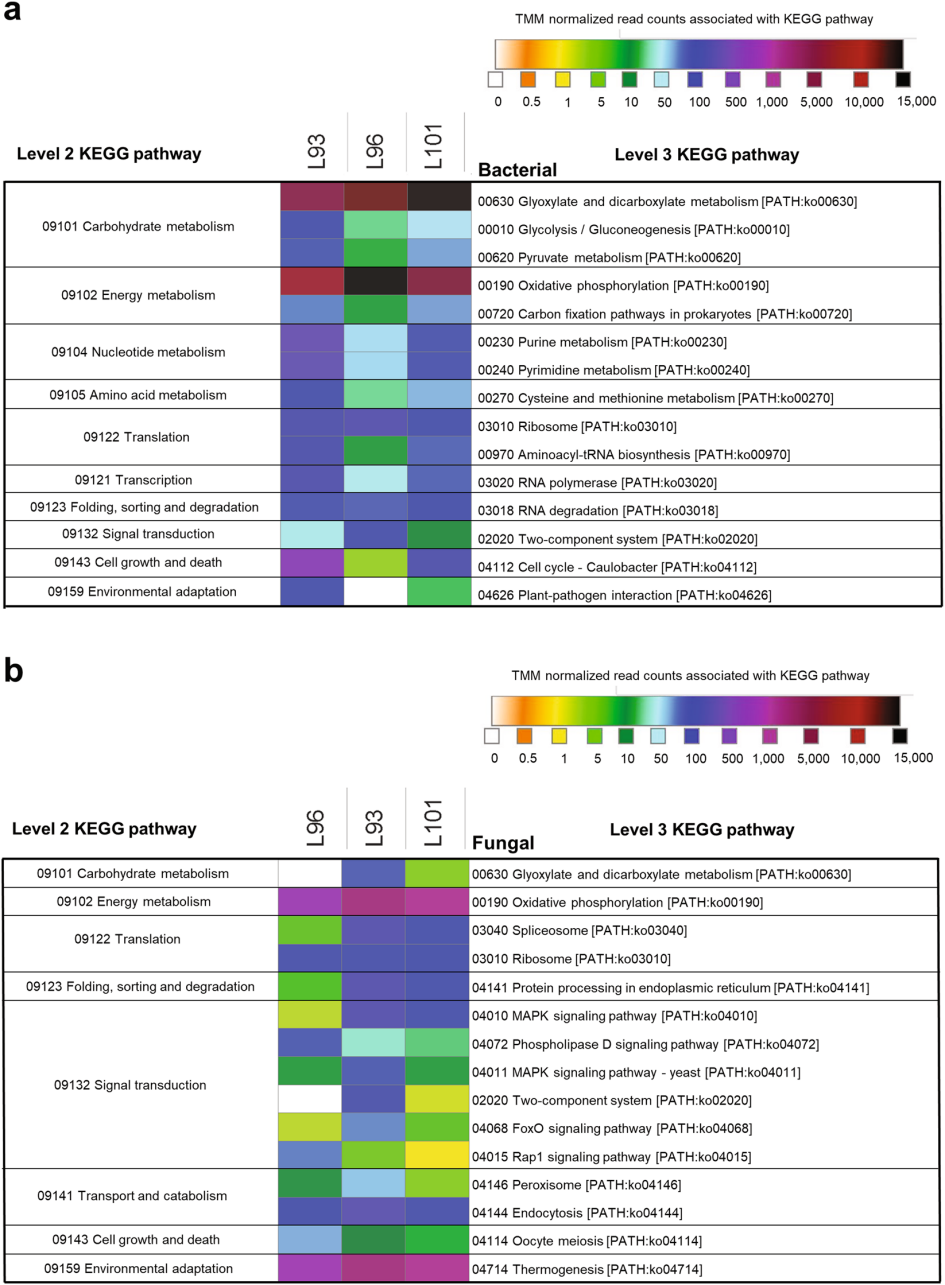
Clustering of the top 15 bacterial and fungal metatranscriptomics data according to their KEGG pathway profiles. Heatmap shows the top 15 distribution of protein-coding reads matching KEGG pathways in the **a** bacterial and **b** fungal community. The color scale shows the TMM-normalized read counts associated with each pathway. Only pathways representing more than five normalized read counts across all three samples are represented. A full version of each of these heatmap can be found in the Supplementary Figs. 2 and 3.

Assessment of the expressed transcripts within the lettuce leaf microbiome revealed a diverse range of functional genes associated with several key metabolic pathways in both the bacterial (Fig. 3a) and fungal (Fig. 3b) populations. Only those genes with a minimum of five transcripts across all samples were included. The full list of genes and their associated KEGG level 3 pathways are listed in Supplementary Figs. 2 and 3. A total of 106 bacterial KEGG level 3 pathways (Supplementary Fig. 2) and a total of 85 fungal KEGG level 3 pathways (Supplementary Fig. 3) were observed.

Within the lettuce-associated bacterial community, the top 15 bacterial KEGG level 3 pathways comprised 94.28% of the reads that were assigned to 106 distinct pathways (Supplementary Fig. 2). The two most dominant metabolisms observed within the lettuce-associated bacterial community transcriptome were the glyoxylate and dicarboxylate metabolism with a mean of 47.64% of the transcripts and oxidative phosphorylation with a mean of 40.33% transcripts mapping to this pathway (Fig. 3a and Supplementary Fig. 2).

As visualized in the fungal metatranscriptome heatmap, the top 15 KEGG level 3 fungal pathways accounted for 84.11% of the total read counts that were assigned to 85 distinct pathways (Fig. 3b, Supplementary Fig. 3, and Supplementary Dataset 2). The two dominant fungal pathways observed in the lettuce-associated microbiome included thermogenesis with a mean of 30.43% of the annotated transcripts and oxidative phosphorylation with a mean of 30.25%. Interestingly, the expression of transcripts associated with several distinct signal transduction pathways was observed including the mitogen-activated protein kinase (MAPK) signaling pathways (4.10%), phospholipase D signaling pathway (1.20%), two-component system (0.74%), FoxO signaling pathway (0.63%), and Rap1 signaling pathway (0.63%) (Fig. 3b and Supplementary Dataset 2).

### Gene network analysis of the expressed transcripts within the lettuce-associated fungal community

**Fig. 4:**
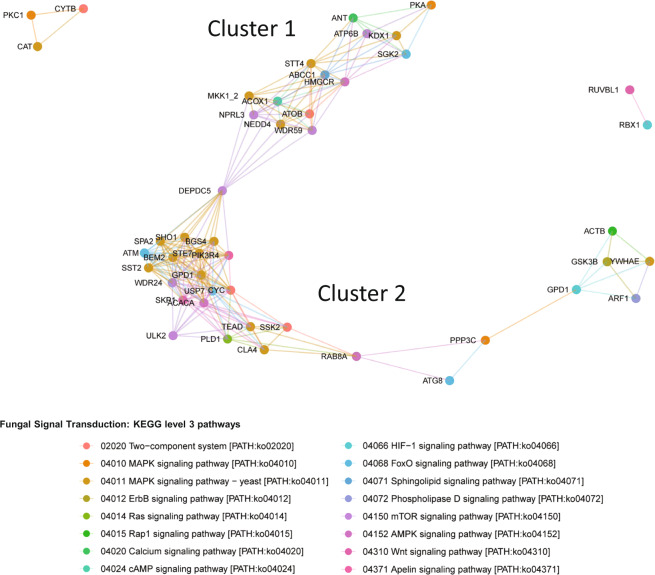
Gene expression network of the fungal KEGG level 2 signal transduction pathway using Bray–Curtis dissimilarity. Each circle represents a KEGG Orthology (KO) gene and lines connect similar expression patterns across the three samples. Only KOs associated with signal transduction pathways within the fungal community are displayed.

The co-expression network within the signal transduction pathway of the fungal community was clustered by KEGG and mapped using the Bray–Curtis dissimilarity (cutoff = 0.5) approach in Phyloseq (Fig. 4). The nodes represent a KEGG Orthology (KO) gene and the lines (edges) represent the connection to similar expression patterns across the three samples. Shorter edges are indicative of closer connections, whereas longer edges show expression patterns that vary more between the samples. Transcripts that are found in only one sample were excluded. Both nodes and edges are color-coded to represent the KEGG level 3 pathways within the overarching KEGG level 2 signal transduction pathway. The gene network map generated showed two main clusters with high levels of connectivity. In cluster 1, genes associated with a diverse set of KEGG level 3 pathways were intertwined and observed to have some similarity in expression patterns across all three samples. The edges connecting each gene in cluster 1 were longer, which showed more variation in expression patterns between each sample. However, cluster 2 showed an enrichment of genes primarily associated with the MAPK signaling pathway that have remarkably similar expression patterns across all three samples as the edges between genes are shorter. This result suggests that the genes in the MAPK signaling pathway within the fungal community may exhibit a coordinated, co-expression pattern.

## Discussion

To prepare for a long-duration spaceflight, it will be critical to develop effective and rapid monitoring tools to evaluate changes in the microbiomes associated with both the spacecraft and the organisms within. In this study, we described such a methodology for analyzing the metatranscriptome of the microbial communities associated with lettuce crops using the nanopore MinION-sequencing platform. Specifically, we have optimized an enrichment protocol and analysis pipeline for microbial transcripts from plant–microbe total RNA using the nanopore MinION, which can serve as a foundation for future onboard plant monitoring. In addition, the results from this study provide a first look into the metatranscriptome of the lettuce microbiome grown under ISS-like environmental conditions.

By understanding the complexity of plant–microbe relationships, the underlying drivers that either maintain or perturb these associations in the space environment can be identified^8^. One challenge, however, is to ascertain how these changes to plant–microbe associations occur across different temporal and spatial scales. Nanopore sequencing has emerged as a viable platform to address these challenges and its applications to monitor changes in microbial communities aboard the ISS using metagenomics are now emerging^34^. However, its applications to examine metatranscriptome changes within a community is relatively new.

One aspect of using nanopore sequencing for metatranscriptome analysis that had to be optimized was the low signal of the native surface and endophytic microbial transcripts from plant–microbial total RNA^41^. In this study, we developed an enrichment protocol for microbial RNAs through the depletion of plant mRNA and rRNA from the lettuce samples. As visualized in Fig. 1, there was an enrichment of the microbial RNA as the depletion of the host RNA resulted in an average loss of ~93% of the total RNA. However, it was evident that there were still residual plant RNA molecules in our library construction (Table 1). The remaining plant RNA was likely attributed to the use of a commercial plant RiboMinus kit that has not been thoroughly optimized for the lettuce species. For instance, although the lettuce 18S ribosomal sequence aligned well with the 18S probe, the incomplete lettuce 28S sequence on the National Center for Biotechnology Information (NCBI) database made it difficult to assess how well the kit would work on lettuce samples.

In addition, the results also demonstrated that for long-term monitoring of specific food crops, species-specific ribosomal probes will need to be designed for host organisms to improve the removal of host RNAs and facilitate the microbial RNA enrichment process. Even though an average of 73.69% of the UniProt-annotated mRNA reads were mostly plant-derived, a substantial number of those reads were associated with cellular components associated with the chloroplast. Due to the similarities between chloroplast RNA and cyanobacteria^42^, these results suggest that removal of plant host organelle sequences through synthetic oligomers^43^ prior to sequencing will be required for optimum enrichment of microbial RNA.

Although this protocol has the potential to be completely conducted in space by the crew, a substantial amount of refinement of our protocols coupled with technology on board the ISS will be needed to make it more practical for being performed in microgravity on a regular basis. These improvements include the automation of molecular protocols, such as sample purification and library preparation with microfluidics. The National Aeronautics and Space Administration, USA, is currently developing tools, such as µTitan^44^, to facilitate autonomous nucleic acid extraction and is also working with companies such Oxford Nanopore Technologies to facilitate autonomous library preparations and magnetic purification with the VolTRAX-v2 hardware in microgravity^45^. Thus, this is the first step of many towards being able to investigate plant–microbial interactions on board the ISS and other low Earth orbit spacecraft. Such capabilities will ultimately advance our understanding of the plant–microbial interactions holistically in the spaceflight environment, which will allow for innovative preventative and proactive care of the crops so as to improve our ability to establish a safe and sustainable space agriculture.

Currently, the sample preparation from plant tissue to library prep for MinION sequencing takes ~8 h and sequencing on the flowcell can run up to 48 h for deeper coverage. The data generated will then be put through the bioinformatics pipeline and followed by data analysis. Hence, depending on the level of detail required, monitoring the functional activity of the plant microbiome with this protocol, from harvesting samples to analyzing the generated sequences, may take ~2–5 days. Future development to optimize and automate this protocol for flight application could shorten the time requirements, which is a stark contrast to current capabilities, as it took ~12 months to determine the primary causal agent of the *F. oxysporum* infection on *Zinnia* plants on board the ISS^27^.

In addition, this initial study provides important insight into the diversity and transcriptional activity of the *L. sativa* cv “Outredgeous” lettuce leaf microbiome grown under ISS-like conditions. Normalized 16S and 18S rRNA reads from each sample were categorized into 43 different phyla (Supplementary Dataset 1) and were consistent across the three biological replicates, which were each run on separate MinION flowcells. Within the top 5 phyla, 39 genera with more than 500 reads in all 3 replicates were highlighted in Fig. 3b and the most transcriptionally active bacterial taxa were *Peptoniphilus* spp. and *Clostridium* spp.

*Peptoniphilus* are Gram-positive anaerobic cocci that uses peptone as a major energy source and produces butyrate as a major metabolic end product^46^. Butanoate metabolism was represented as part of the carbohydrate metabolism in the full bacterial heatmap (Supplementary Fig. 2). *Peptoniphilus* were also found to be one of the most abundant phyllosphere taxa found on tomato leaf samples^47^ and are, therefore, common taxa in plant–microbe interactions.

*Clostridium*, the other highly transcriptionally active taxa, is an extremely heterogeneous genus of bacteria that is ubiquitous in a wide range of environments, such as soil, sewage, and the intestines of humans and animals^48,49^. Some *Clostridium* species, such as *Clostridium botulinum*, are known pathogens that can form vegetative cells under certain conditions, which produce a fatal toxin to humans^50,51^. However, *Clostridium* species are commonly found on lettuce leaves and can be efficiently removed through proper washing of the leaves before consumption^51^. Therefore, these results again reinforce the importance of regular monitoring of the transcriptional and metabolic activity of taxa within plant growth habitats during spaceflight.

The taxonomic results derived from this developed method differed from previous metagenomic analyses on the lettuce leaf microbiome and reinforce the importance of conducting on-site analyses of the plant microbiome^17^. For example, neither genera *Peptoniphilus* or *Clostridium* were observed in the previous metagenomic analysis^17^. In that study, the top five recovered taxa were *Bacillus*, *Burkholderia*, *Macrococcus*, *Methylobacterium*, and *Staphylococcus*, which were observed in our metatranscriptome data (Fig. 2b), but at lower relative abundances. Interestingly, *Thermogemmatispora*, which was their most abundant genera in almost all their samples, and *Pelomonas* were not detected in our dataset. These differences in the taxonomy diversity results between the metatranscriptomic and metagenomic approaches are not unexpected. Metagenomic analysis are based on DNA molecules, which could come from non-viable microorganisms^52^, whereas the metatranscriptomic approach based on the RNA molecules provides insight into the functionally active microbial community. Despite the differences in the two approaches, there were still substantial overlaps in the taxonomic composition of the lettuce microbiome, especially for the bacterial community, suggesting the nanopore sequencing of the metatranscriptome was highly complementary to the previous metagenomic studies on lettuce microbiome^17^.

Within the observed fungal community, the most transcriptionally active taxa were *Fusarium* spp. and *Colletotrichum* spp. (Fig. 2b). Both these genera are known to have species with strains that are plant pathogens. These cosmopolitan fungi have a broad range of hosts and can cause wilt diseases and anthracnose attacks in many major agricultural crops^53–55^. However, as there are many species and strains within each of these genera of fungi that are not pathogenic, the specific ecological role of the recovered taxa could not be fully assessed. The use of this nanopore metatranscriptomic sequencing approach could be used to prioritize samples and taxa for downstream in-depth sequencing, to ascertain the pathogenic potential, if any, of recovered fungal taxa.

The metatranscriptomic analyses of the transcribed functional genes of the bacterial and fungal community were largely consistent with the core functional microbiome in other phyllosphere microbial systems reported in the literature^56,57^. In our study, within the top 15 most actively transcribed bacterial pathways (Fig.3a), “Glyoxylate and dicarboxylate metabolism” had 47.64% of the total reads and “Oxidative phosphorylation” had 40.33% of the total reads. This result was not unexpected, because glyoxylate metabolism is known to be involved in the metabolism of short carbon compounds (one or two carbons), which are an important energy source for leaf-associated bacteria^58^. Oxidative phosphorylation is a vital part of metabolism, as the end product is adenosine triphosphate and is required for a myriad of cellular processes.

Most of the top bacterial pathways listed in our study (Fig. 3) are typically involved in essential housekeeping activities and are necessary for bacterial survival. However, signal transduction and environmental adaptation pathways could provide some indication as to how the plants are responding to their environment. For example, the high number of reads that mapped to the two-component system pathway might suggest the need to sense and quickly respond to environmental factors by phosphorylating and dephosphorylating proteins, whereas the plant–pathogen interaction pathway could indicate a biotic stress response^59^.

In the fungal metatranscriptome, “Oxidative phosphorylation” and “Thermogenesis” were the most actively transcribed pathways. In addition to oxidative phosphorylation, the mitochondria also engage in thermogenesis, which uncouples the oxidative phosphorylation for heat production in organisms^60^. However, intriguingly, about a quarter of all the KEGG level 3 pathways listed (Supplementary Fig. 3) were associated with the signal transduction pathway. Many of these were also seen in the top 15 most actively transcribed fungal pathways (Fig. 3b). The most actively transcribed signal transduction pathway in the fungal metatranscriptome was the MAPK signaling pathway (Fig. 3b). The MAPK pathway plays important roles in fungal physiology and development, as it governs diverse growth processes such as mating, cell cycle control, morphogenesis (e.g., filamentation), response to different stresses, cell wall assembly and integrity, pathogenicity, cell to cell signaling, fungal–plant interactions, and response to damage-associated molecular patterns^61–63^.

From our metatranscriptomic dataset, we can map genes expressed to specific processes within the MAPK signaling pathway in yeast (Supplementary Fig. 4). This result suggests that the fungi within the lettuce microbiome may be actively engaged in cell wall remodeling and proliferation. Moreover, the gene network mapping of the signal transduction transcripts showed high levels for connectivity of genes (i.e., *SST2*, *STE7*, *GDP1*, *SHO1*, and *CLA4*) within the MAPK pathways, which indicates that these are tightly connected processes in the fungal community (Fig. 4). Interestingly, many of these genes also overlap with fungal pathogenesis^61,64^ and, although this does not necessarily indicate active pathogenicity, it would be beneficial for the crew to monitor these genes over time in their food crops.

Currently, many of the commercially available portable rapid plant disease diagnostic tools are based on immunodetection methods (enzyme-linked immunosorbent assays), PCR-based assays, and spectrometry-based protocols, which require some prior prediction of the microbial organisms that are being tested, to design antibodies or PCR primers for the analyses^65,66^. The MinION platform has great advantages when dealing with diagnosing plant pathogens and plant–microbial analysis, as it uses shotgun sequencing technology that requires no prior knowledge or prediction about the possible causative agent. The detection of any microbial organisms, even at low levels, is limited to the microbial sequences available in the NCBI database.

Although this study described the analysis of the lettuce microbiome at a single time point, the methodology developed in this study has the potential to enable the comparative analyses of the lettuce microbiome across spatial and temporal scales to identify potential drivers of change during plant growth and development. The complementary use of rapid nanopore-sequencing approaches with additional more in-depth metagenomic sequencing of viable microorganisms will enable baselines of health to be regularly monitored in plant growth habitats aboard spacecraft. These comparisons will also enable more refined approaches to understand how the microbiome changes under environmental perturbations and contributes to healthy plant growth and development during a long-duration spaceflight.

## Methods

### Sample collection and RNA extraction

Seeds of red romaine lettuce, *L. sativa* cv “Outredgeous” were surface-sanitized with a bleach-hydrochloric acid solution (30 ml 6% Pure Bright Germicidal Ultra Bleach and 500 μl 37% hydrochloric acid) for 1 h and allowed to off-gas for 24 h prior to planting. Sanitization was verified by placing seeds on trypticase soy agar and inhibitory mold agar plates, and incubating for 48–72 h at 30 °C^18^. A standard validation protocol was used to conform the effectiveness of the sanitization protocol^26^. No bacterial or fungal growth was observed on the plates during the screening process. Three surface-sanitized seeds were individually placed in 4 inch pots containing greens-grade arcillite, a calcined clay and time-release fertilizer typically used in spaceflight hardware^26^, and were grown in environmentally controlled growth chambers at the KSC that mimicked conditions of the ISS. The growth chambers were cleaned to remove any potential human-associated microbes before use, but were not sterilized.

To simulate conditions aboard the ISS, the plants were maintained at 50% relative humidity, 3000 p.p.m. CO_2_, and 23 °C. The growth cycle was established on a 16 h (light)/8 h (dark) cycle under fluorescent cool white lights (200–300 μE m^−2^ s^−1^) and all were on daily automatic watering with deionized water. Pots were thinned to one plant each at day 7 and samples were harvested at day 28 for leafy greens. Lettuce leaf tissue from each of the three pots (denoted as L93, L96, and L101) was placed immediately into RNAlater (Sigma-Aldrich, St. Louis, MO) and stored at −80 °C until ready for RNA extraction.

Total RNA was extracted from each of the three lettuce leave replicates with the RNeasy Plant Mini Kit following the manufacturer’s protocol (Qiagen, Valencia, CA). The recovered RNA was DNase-treated with the optional on-column digestion following the manufacturer’s protocol (Qiagen, Valencia, CA) and quantified using Qubit 2.0 (Thermo Fisher Scientific, Waltham, MA). A sample of clean RNAlater was used as a negative control throughout the sample preparation process. No RNA was obtained or amplified from the negative control sample.

### Enrichment of microbial transcripts

An enrichment method was developed to enable the separation of plant and microbial RNAs for subsequent sequencing with the Oxford Nanopore-sequencing technology^41^. Using the recovered host–microbial total RNA (7.33–23.56 µg), the Dynabeads poly(A) capture protocol (Thermo Fisher Scientific, Waltham, MA) was performed to remove poly(A)-containing RNAs (e.g., plant mRNAs). Of note, plants and fungi both have poly(A)-containing mRNA. It is therefore likely that some of the fungal RNA were also removed during the enrichment process. The remaining poly(A)-depleted material (microbial mRNA/rRNA and plant rRNA) was then purified with the Zymo RNA clean and concentration kit according to the manufacturer’s protocol (Zymo Research, Irvine, CA). The remaining RNAs were processed using the RiboMinus Plant Kit for the removal of plant rRNA as per the manufacturer’s protocol (Thermo Fisher Scientific, Waltham, MA). The host-depleted RNA (1–10 μg) from each sample was then A-tailed using Poly(A) polymerase (New England Biolabs, Ipswich, MA) for 25 min at 37 °C. The enriched microbial RNA (mRNA and rRNA) quantity was assessed with a Qubit 2.0 fluorometer (Thermo Fisher Scientific, Waltham, MA) and quality was assessed using a 2100 Bioanalyzer (Agilent Technologies, Inc., Santa Clara, CA). To assess the purity of RNA, A260/280 readings were also taken with the UV-1800 Shimadzu spectrophotometer (Shimadzu, Kyoto, Japan). The samples were stored at −80 °C until library preparation.

### Nanopore cDNA-PCR library preparation

Library preparation for cDNA-PCR sequencing was performed using the SQK-PCS109 sequencing kit, following the manufacturer’s instructions (Oxford Nanopore Technologies, Oxford, UK). Briefly, 50 ng of enriched microbial RNA samples from L93, L96, and L101 were reverse transcribed using Maxima H Minus Reverse Transcriptase (Thermo Fisher Scientific, Waltham, MA) and incubated in a thermal cycler at 42 °C for 90 min and then 85 °C for 5 min. To amplify the products without introducing biases, full-length transcripts were selected using the Nanopore rapid attachment primers (SQK-PCS109) using LongAmp Taq 2× Master Mix (New England Biolabs, Ipswich, MA, USA) with the following cycling conditions, an initial denaturing step at 95 °C for 5 min, followed by 14 cycles of 15 s at 95 °C, 15 s at 62 °C, and 1 min at 65 °C, and a final extension step at 65 °C for 6 min and a hold at 4 °C. The products were then purified and separated using Agencourt AMPure XP beads (Beckman Coulter, Indianapolis, IN) with 1.8 (v/v) ratio of magnetic beads to the reaction mixture. The cDNA library using 100 fmol of amplified cDNA with adapters was loaded onto a FLO-MIN106D R9 flowcell and sequencing was performed with a MinION device for 48 h. cDNA derived from each replicate (L93, L96, L101) were sequenced on individual flowcells to provide information about the process reproducibility and variability.

#### Bioinformatics and data analysis

RNA-sequencing data were base-called in real-time using an integrated version of Guppy in the MinKNOW software v3.3.2 via the MinIT device v19.05.2. All FASTQ files were concatenated and used in the bioinformatics pipeline illustrated in Supplementary Fig. 1. Briefly, MinION-sequencing statistics were analyzed using Nanoplot v1.0.0^67^ and adapters were trimmed using Porechop v0.2.4^68^. Reads were filtered for a minimum read length of 100 bp and minimum *Q*-score of 5 using filtlong v0.2.0 was to ensure that >80% of the reads in all three samples were as previously described^69^. Large chimeric reads of >30,000 bp were also filtered out using a Unix shell command. The N50 read length, which is defined as the read length such that reads of this length or greater composes at least 50% of the total bases, was 417, 616, and 512 bp for the L93, L96, and L101 samples, respectively, whereas the average read length across all samples was 443 bp (Table 1).

To remove any residual host plant transcripts and rRNA, reads were mapped to well-assembled genomes within the Asteraceae family (e.g., lettuce and sunflower) using Bowtie2^70^, then screened with SortMeRNA v2.1^71^ and removed. Taxonomic classification of microbial 16S and 18S rRNA reads was performed with Kraken2 v2.0.8b^72^ using the kraken2-microbial database (https://lomanlab.github.io/mockcommunity/mc_databases.html) derived from the NCBI Reference Sequence database (release 89)^73^. Reads counts were normalized across all three samples and only operational taxonomic units with >500 assigned reads were included and visualized with bubble plots created using the R package ggplot2 v3.3.0^74^.

The remaining metatranscriptome sequences were annotated with Trinotate v3.0 (http://trinotate.github.io) using BLAST + v2.9.0^75^ against the Uniprot database^76^. No assembly was performed on the reads before the annotation, as only high-quality long reads were used in the downstream analyses; this approach has previously been used to annotate MinION-derived reads^77,78^. Apart from fungi, coding sequences assigned to viruses and other eukaryotes were excluded in the downstream analysis so as to focus on the microbial activity found associated with the lettuce leaf. In addition, transcripts derived from taxa assigned to Cyanobacteria, which represented a substantial number of reads (Supplementary Dataset 1), as well as transcripts associated with photosynthesis genes (Supplementary Dataset 2), were excluded to prevent the inclusion of possible misassigned chloroplast transcripts. Abundance of transcripts that had KEGG annotations^79^ were normalized using gene-length corrected transcripts per million method followed by Trimmed Mean of *M*-values method as implemented in EdgeR^80^. KEGG-annotated association with their respective pathways were performed using the R package KEGGREST^81^. Heatmaps were generated using the Broad Institute’s webtool Morpheus (https://software.broadinstitute.org/morpheus). The hierarchical clustering of the full heatmaps were done using the one minus Pearson’s correlation metric in Morpheus. The gene networking analysis was completed using the R package Phyloseq v1.19.1^80,82^.

### Reporting summary

Further information on research design is available in the Nature Research Reporting Summary linked to this article.

## Supplementary information

Supplementary Information

Supplementary Data 1

Supplementary Data 2

Reporting Summary

## Data Availability

The raw and processed MinION Oxford Nanopore-sequencing data reported in this study have been deposited with NCBI at Gene Expression Omnibus (GEO) repository (GSE152914) and with NASA’s GeneLab (GLDS-307).
