# Supplementary material for: Evaluating the lettuce metatranscriptome with MinION sequencing for future spaceflight food production applications
Source: NPJ Microgravity. 2021 Jun 17;7:22. doi: 10.1038/s41526-021-00151-x (PMC8211661; doi:10.1038/s41526-021-00151-x)
Supplement: Supplementary file 1 — Supplementary Information [file 41526_2021_151_MOESM1_ESM.pdf]

Evaluating the lettuce metatranscriptome with MinION sequencing for future spaceflight food production applications

Natasha J. Sng-Haveman[1], Christina L.M. Khodadad[2], Anirudha R. Dixit[2], Artemis S. Louyakis[3], Gioia D. Massa[4], Kasthuri Venkateswaran[5], Jamie S. Foster[1*]

[1]Microbiology and Cell Science, University of Florida, Space Life Science Lab, Merritt Island Florida, USA.

[2]Amentum Services, Inc., LASSO, Kennedy Space Center, Merritt Island, Florida, USA.

[3]Molecular and Cell Biology, University of Connecticut, Storrs, Connecticut, USA.

[4]Space Crop Production Team, Kennedy Space Center, Florida, USA.

[4,5]Biotechnology and Planetary Protection Group, Jet Propulsion Laboratory, Pasadena, California, USA.

Running title: Metatranscriptomics of food crop microbiomes using MinION

*Corresponding Author: jfoster@ufl.edu

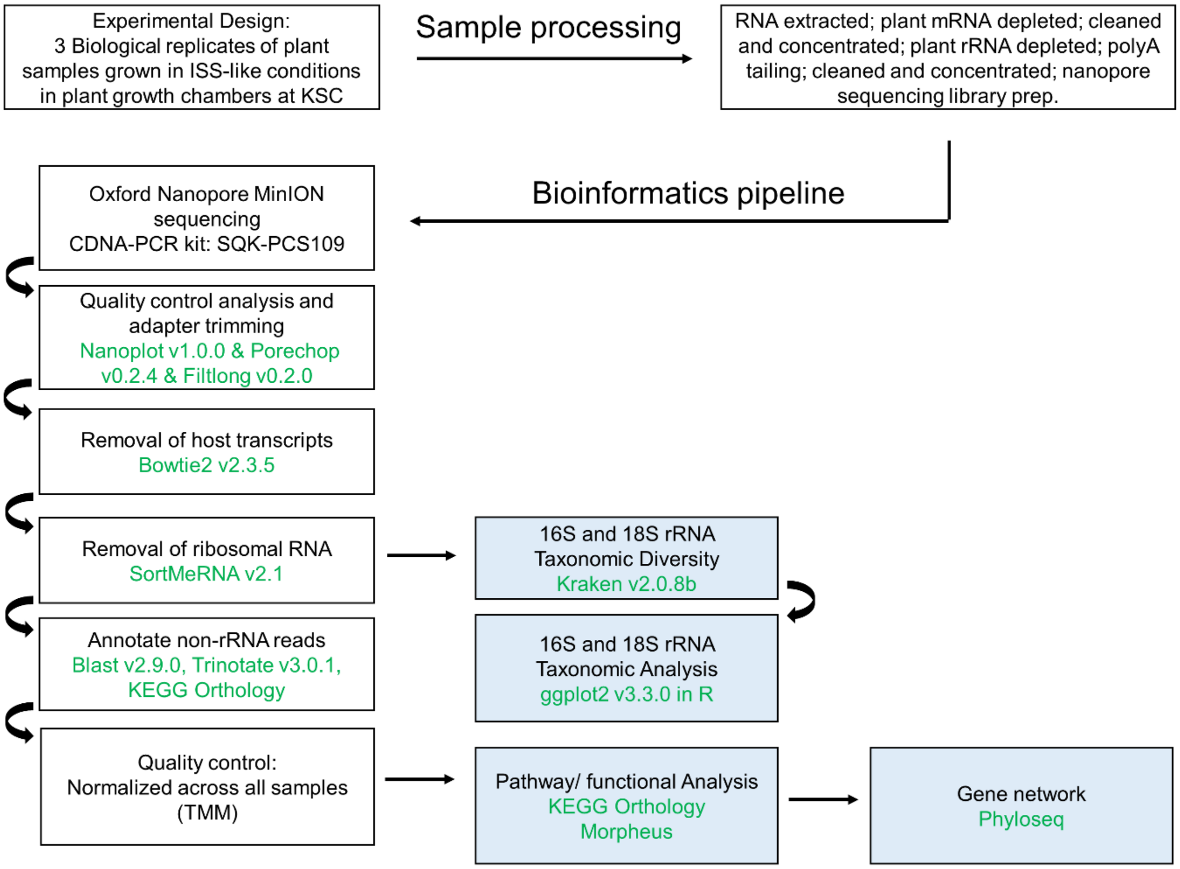

Supplementary Fig. 1. Experimental and bioinformatics pipeline for analyzing the plant microbiome metatranscriptome.

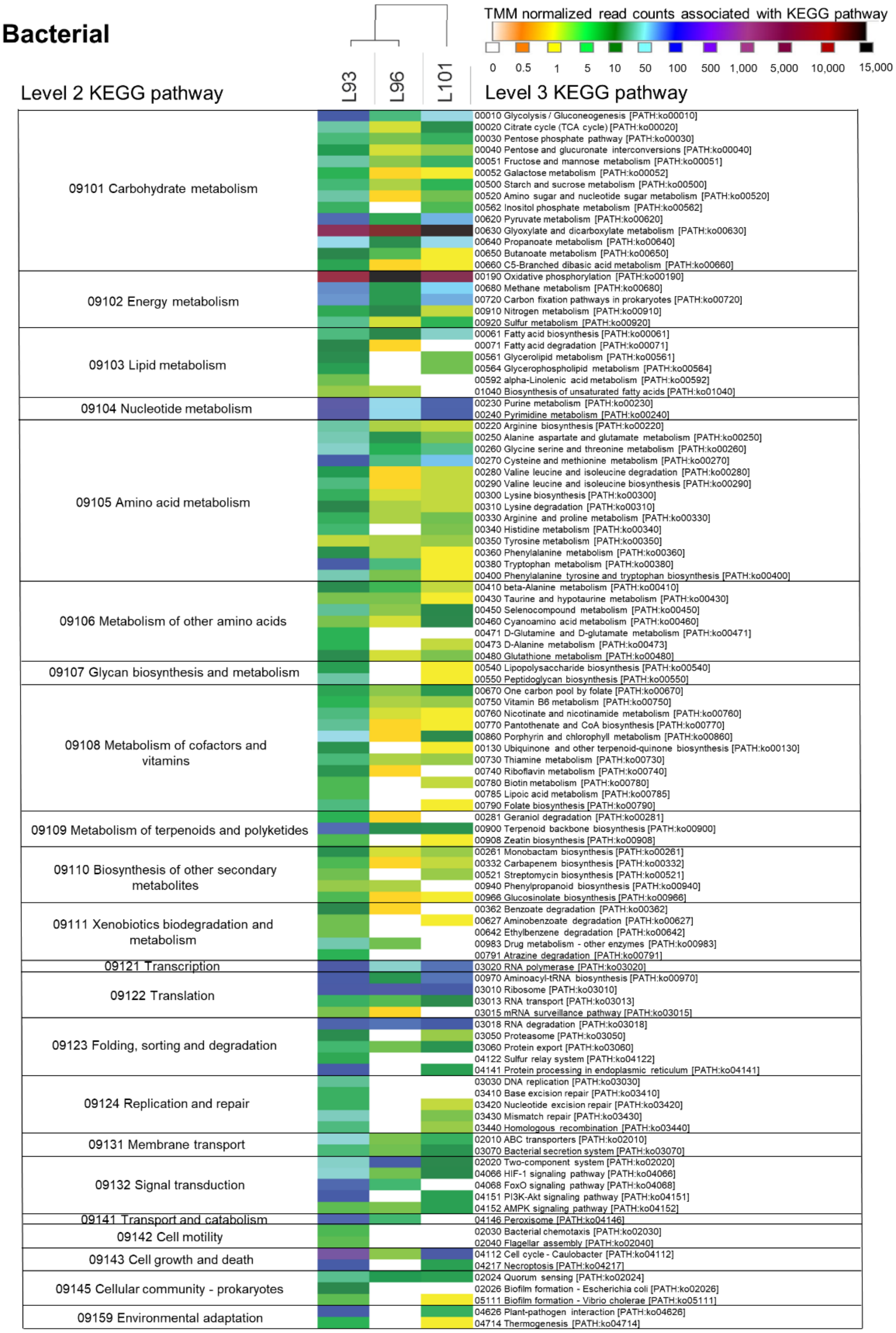

Supplementary Fig. 2: Clustering of the full bacterial metatranscriptomics data according to their KEGG pathway profiles. Heatmap shows relative distribution of protein-coding reads matching KEGG pathways. The color scale shows the TMM normalized read counts associated with each pathway. Only pathways representing more than 5 mapped reads across all three samples are represented.

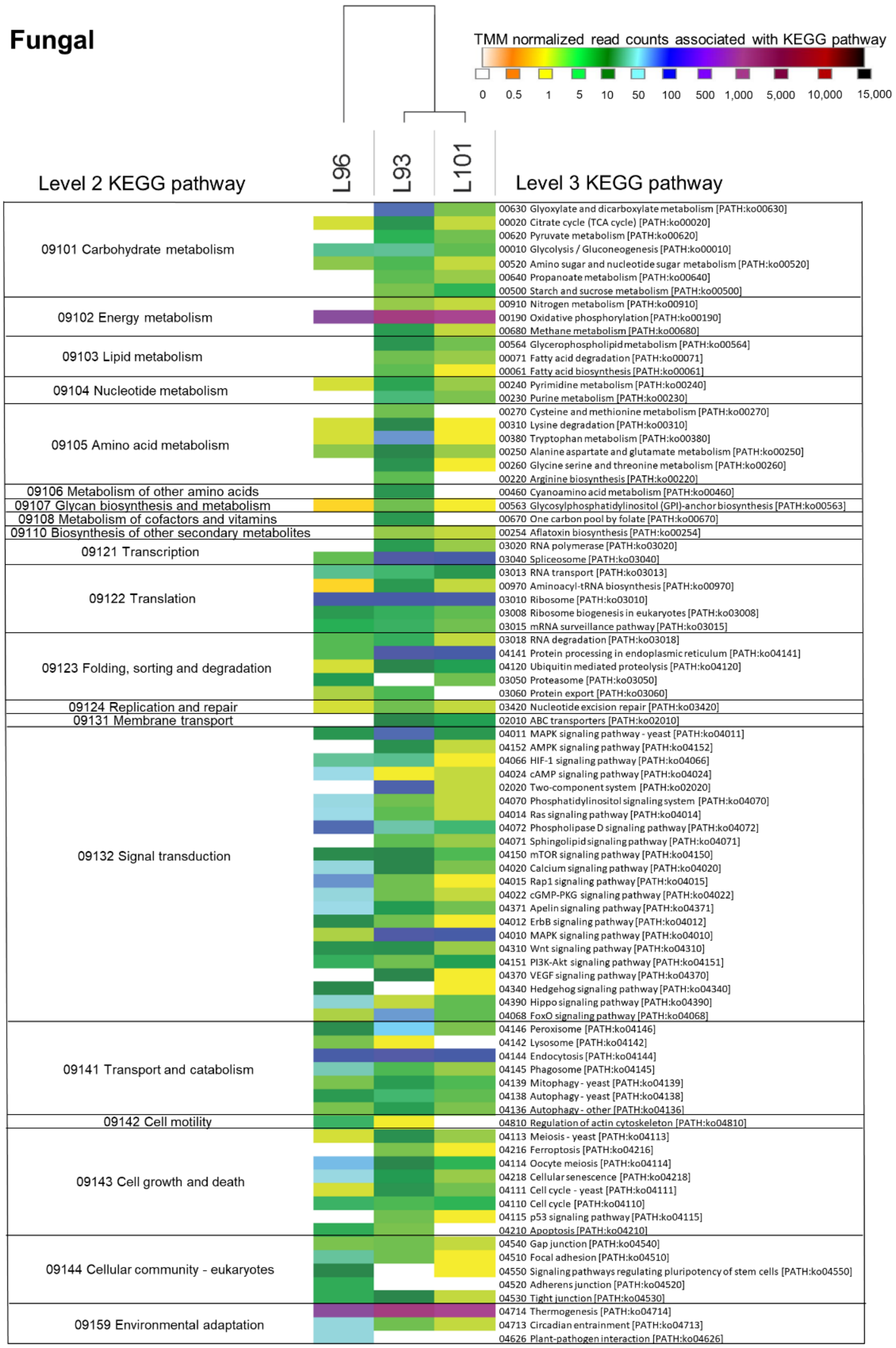

Supplementary Fig 3. Clustering of the full fungal metatranscriptomics data according to their KEGG pathway profiles. Heatmap shows relative distribution of protein-coding reads matching KEGG pathways. The color scale shows the TMM normalized read counts associated with each pathway. Only pathways representing more than 5 mapped reads across all three samples are represented.

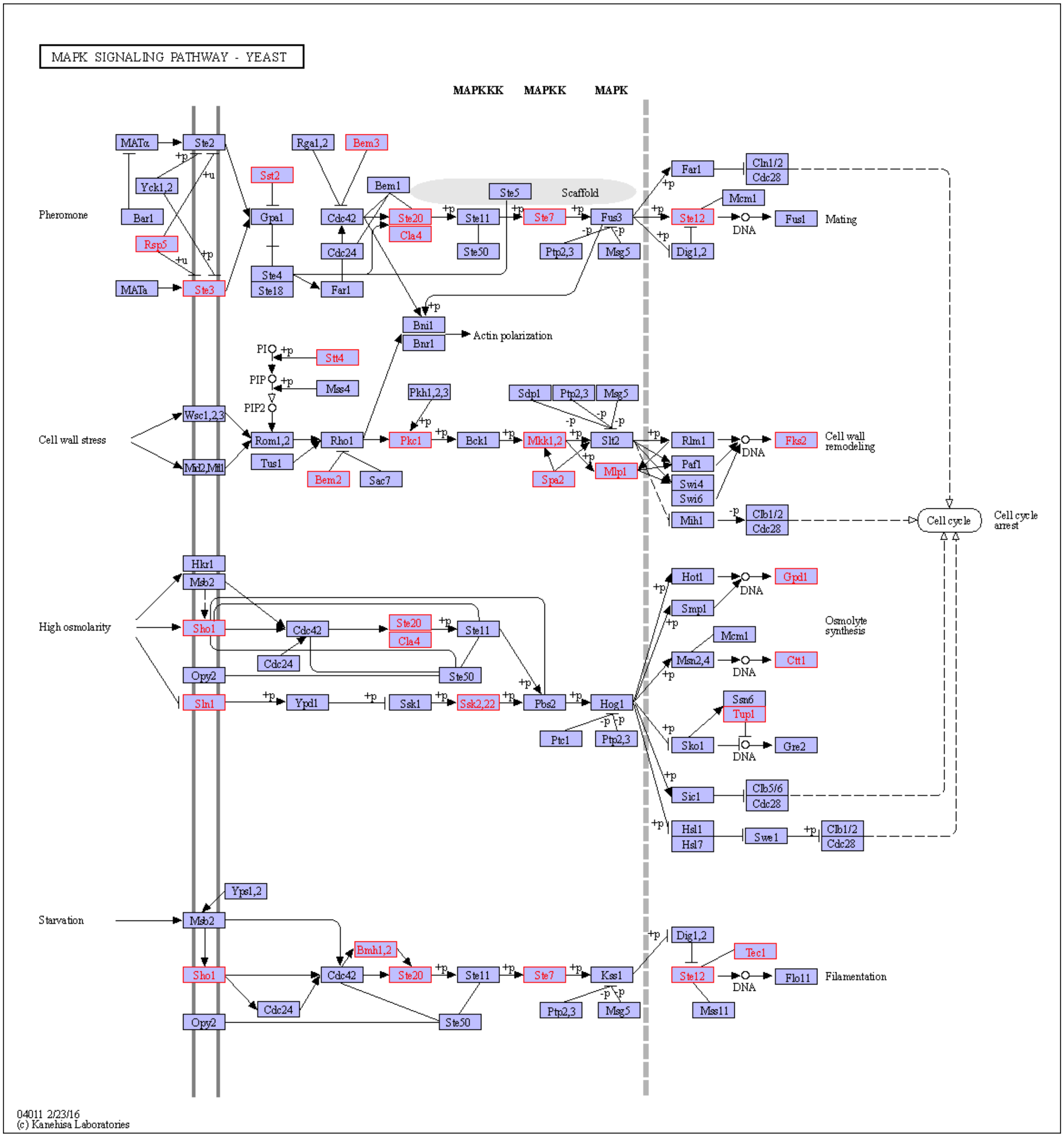

Supplementary Fig. 4. Level 4 KEGG genes within the fungal metatranscriptomic dataset. The genes highlighted in red were found in our metatranscriptomics analysis. Each gene within our dataset is mapped to the specific processes associated with the MAPK signaling pathway. This map was generated from the KEGG orthology database website (https://www.genome.jp/kegg/ko.html).

Supplementary Dataset 1. Raw, normalized, and filtered read counts for taxonomic analyses used in this study.

Supplementary Dataset 2. Bacterial and fungal KEGG Orthology (KO) and read count data used for functional activity analysis.
